# Virtual Reality Therapy for Adults Post-Stroke: A Systematic Review and Meta-Analysis Exploring Virtual Environments and Commercial Games in Therapy

**DOI:** 10.1371/journal.pone.0093318

**Published:** 2014-03-28

**Authors:** Keith R. Lohse, Courtney G. E. Hilderman, Katharine L. Cheung, Sandy Tatla, H. F. Machiel Van der Loos

**Affiliations:** 1 School of Kinesiology, Auburn University, Auburn, Alabama, United States of America; 2 School of Kinesiology, University of British Columbia, Vancouver, British Columbia, Canada; 3 Department of Physical Therapy, University of British Columbia, Vancouver, British Columbia, Canada; 4 Department of Occupational Science and Occupational Therapy, University of British Columbia, Vancouver, British Columbia, Canada; 5 Department of Mechanical Engineering, University of British Columbia, Vancouver, British Columbia, Canada; University of Glasgow, United Kingdom

## Abstract

**Background:**

The objective of this analysis was to systematically review the evidence for virtual reality (VR) therapy in an adult post-stroke population in both custom built virtual environments (VE) and commercially available gaming systems (CG).

**Methods:**

MEDLINE, CINAHL, EMBASE, ERIC, PSYCInfo, DARE, PEDro, Cochrane Central Register of Controlled Trials, and Cochrane Database of Systematic Reviews were systematically searched from the earliest available date until April 4, 2013. Controlled trials that compared VR to conventional therapy were included. Population criteria included adults (>18) post-stroke, excluding children, cerebral palsy, and other neurological disorders. Included studies were reported in English. Quality of studies was assessed with the Physiotherapy Evidence Database Scale (PEDro).

**Results:**

Twenty-six studies met the inclusion criteria. For body function outcomes, there was a significant benefit of VR therapy compared to conventional therapy controls, G = 0.48, 95% CI = [0.27, 0.70], and no significant difference between VE and CG interventions (P = 0.38). For activity outcomes, there was a significant benefit of VR therapy, G = 0.58, 95% CI = [0.32, 0.85], and no significant difference between VE and CG interventions (P = 0.66). For participation outcomes, the overall effect size was G = 0.56, 95% CI = [0.02, 1.10]. All participation outcomes came from VE studies.

**Discussion:**

VR rehabilitation moderately improves outcomes compared to conventional therapy in adults post-stroke. Current CG interventions have been too few and too small to assess potential benefits of CG. Future research in this area should aim to clearly define conventional therapy, report on participation measures, consider motivational components of therapy, and investigate commercially available systems in larger RCTs.

**Trial Registration:**

Prospero CRD42013004338

## Introduction

Stroke is a leading cause of death and disability around the world, and the majority of survivors experience chronic motor deficits associated with reduced quality of life [1]. Neurophysiological data suggest considerable amounts of practice are required to induce neuroplastic change and functional recovery of these motor deficits [2]–[4]. This requisite high repetition is problematic, however, because observational data show that clients generally perform a very limited number of movement repetitions in traditional therapy sessions [5]. Furthermore, many logistical, financial, environmental, and individual barriers limit the efficacy of conventional therapy for adults post-stroke [6],[7]. Consequently, research is often focused on optimizing an individual's potential amount of recovery for a given amount of time in therapy. One proposed method for optimizing the effects of therapy is the use of virtual reality (VR). VR can be defined as a type of user-computer interface that implements real-time simulation of an activity or environment allowing user interaction via multiple sensory modalities [8]. VR therapies are an appealing avenue of research because they can provide patients and therapists with additional feedback during therapy, increase patient motivation, and dynamically adjust the difficulty of therapy [9]–[11].

Increasingly, VR therapies have been compared to "usual care" or "conventional therapy" (CT) as sophisticated technologies have become more readily available and affordable. VR therapy refers to a broad class of interventions, but can generally be defined as technological interventions that alter properties of the physical world. These properties might be perceptual, such as providing clients with additional sensory feedback about their movement in a virtual environment (VE). At times, VE training is integrated with exogenous forms of support such as robotic assistance or resistance [12],[13], but we restricted our review to interventions that did not include robotic assistance. Moreover, the advent of movement-controlled videogames such as the Wii (Nintendo), Move (Sony), and Kinect (Microsoft) has also allowed therapists to integrate commercial gaming (CG) systems into therapy. Although only a small number of randomized controlled CG studies exist [14]–[17], CG research is appealing because these interventions offer some of the benefits of VE interventions [18], but have greater availability and a significantly reduced cost. Thus, a major objective for the current review was to quantitatively explore the effectiveness of VE and CG interventions compared to CT.

Previous reviews comparing VR therapy to CT exist [19]–[21], and while they indicate moderate positive benefits of VR therapy, overall there is considerable variability in the observed effects. Potential sources of variability include the type and parameters of intervention, the type of outcome being measured, and the demographics of clients being studied, such as the time from stroke to intervention onset and the initial severity of the motor deficit. This review adds to the current body of knowledge about VR therapy by: (1) including new data comparing VR therapies to CT control groups; (2) exploring how VR therapies affect different outcomes according to the International Classification of Function, Disability, and Health (ICF); and (3) exploring how different types of VR therapy affect outcomes, or more specifically, how custom-built VE systems compare to interventions using CG technology.

## Methods

Prior to data collection, the review was registered with the Prospero registry for systematic reviews (#CRD42013004338; http://www.crd.york.ac.uk/NIHR_PROSPERO/). Objectives were defined according to a PICO model (Population, Intervention, Comparison, Outcome). The population of interest was adults post-stroke. Interventions considered were VR therapies that did not include exogenous stimulation (such as functional electrical stimulation) or robotic assistance. Comparison groups included "usual care", "standard care" or "conventional therapy", and could involve physical therapy (PT) and/or occupational therapy (OT). (See Table 1 for a description of control therapies.) Primary and secondary outcomes from all studies were considered, provided that these outcomes were behavioural assessments in one of the ICF domains (i.e., body structure, body function, activity, participation). Self-report measures such as the Motor Activity Log (e.g., Housman et al. [22]) or the ABILIHAND inventory (e.g., Piron et al. [23]) were excluded. Restricting our analysis to behavioural measures of function or impairment that compared VR and conventional therapy makes these outcomes more comparable for the purpose of meta-analysis. Further stratifying these results by ICF classification increases comparability, however there are still concerns about differences in the types of CT provided in control groups. These concerns are discussed below.

**Table 1:** Characteristics of trials comparing virtual reality therapy to conventional therapy in adults post-stroke.

Reference	Intervention	VR Intervention	Ctrl Intervention	VR Type	Extracted Outcomes	Outcome Classification
Broeren, 2008 [32]	VE training + CT vs. CT*	3-D computer games with UL unsupported, with rehabilitation personnel	Creative crafts, social and physical activities at activity centre.	VE	BBT, movement time, hand-path ratios	ACT, BF, BF
Cho, 2013 [36]	VE walking + standard therapy vs. CT + standard therapy	Virtual walking training program with video recording, Co-intervention: Standard therapy: Therapeutic exercise, functional therapy, OT, FES	Treadmill gait training Co-intervention: Standard therapy: Therapeutic exercise, functional therapy, OT, FES	VE	BBS, TUG	ACT, ACT
Cikajlo, 2012 [37]	VE balance training vs. CT	VR supported balance training in standing frame, (2 week in clinic & 1 week in home) with PT supervision.	Balance training without VR (in clinic only).	VE	BBS, TUG, 10mWT	ACT, ACT, ACT
Crosbie, 2012 [38]	VE therapy vs. CT	VR tasks focused on UL reaching and grasping with therapist.	Standard UL therapy, including muscle facilitation, stretching, strengthening and functional tasks with PT.	VE	Mobility Index, ARAT	BF, ACT
da Silva Cameirao, 2011 [39]	VE game + Standard Therapy vs. CT + Standard Therapy	Rehabilitation gaming system targeting UL speed, range of motion, grasp and release. Co-intervention: Standard OT & PT.	One of two treatments: 1) Pure occupational therapy targeting object displacement, grasp, and release; or 2) Wii games. Co-intervention: Standard OT & PT.	VE	Mobility Index, FMA, CAHAI	BF, BF, ACT
Gil-Gómez, 2011 [15]	Wii balance board therapy vs. CT	Easy balance VR system with Wii balance board (eBaViR).	Traditional rehabilitation balance exercises individually or in group)	CG	BBS, BBA	ACT, ACT
In, 2012 [40]	VE + Standard Therapy vs. Sham + Standard Therapy	VR reflection therapy for UL movements (with caregiver).	UL movements using unaffected limb (no VR component) (with caregiver).	VE	FMA, BBT, JTHF	BF, ACT, PART
Jung, 2012 [30]	VE treadmill vs. treadmill	VR (with head mounted device) treadmill training.	Treadmill training.	VE	TUG	ACT
Katz, 2005 [41]	VE street-crossing vs. visual training	Desktop VR street-crossing cognitive training.	Computer-based visual scanning tasks.	VE	FIM, VR-performance, Real street crossing.	ACT, ACT, PART
Kihoon, 2012 [34]	VE + Standard Therapy vs. CT*	Interactive Rehabilitation & Exercise System (IREX) VR targeting UL and visual impairments.	Traditional therapy (unspecified).	VE	WMFT, MVPT	ACT, BF
Kim, 2009 [33]	VE + CT vs. CT*	IREX VR balance therapy + CT.	Standard PT, involving neurofacilitation.	VE	BBS, MMAS, 10mWT	ACT, ACT, ACT
Kim, 2012 [14]	Wii games vs. no gaming	Nintendo Wii for balance and motor control + general exercise (unspecified) and electrical stimulation before each session.	General exercise (unspecified) and electrical stimulation before each session.	CG	FIM, PASS, MASS	ACT, BF, BF
Kiper, 2011 [42]	VE therapy vs. CT	Virtual Reality Rehabilitation System (VRRS) training targeting UL functional tasks (turning, pouring, using a hammer, etc.) with PT.	Traditional neuromotor rehabilitation (postural control, in-hand manipulation, fine motor control and coordination) with PT.	VE	FIM, FMA, MAS	ACT, BF, BF
Kwon, 2012 [35]	VE + CT vs. CT*	IREX VR UL training with OT + CT.	Routine OT & PT (gait & balance training, tabletop activities, UL strengthening and functional tasks.	VE	FMA, MFT, MBI	BF, ACT, ACT
Lam, 2006 [43]	VE skills training vs. CT vs. no treatment	2-D VR program targeting various cognitive functions over 10 sessions.	Psychoeducational training (instruction + video modeling) over 10 sessions.	VE	Behavioural assessment of mass transit skills.	PART
Mirelman, 2010 [44]	VE training vs. Non-VE training	Rutgers ankle rehabilitation system (robotic gait training with VR stimulation), involving various ankle movements, with therapist.	Ankle movements without VR under therapist supervision.	VE	Gait speed, ankle movement, ankle power	ACT, BF, BF
Piron, 2007 [45]	VE therapy vs. CT	Reinforced feedback in VR environment for UL training with PT.	Conventional UL therapy (unspecified) with PT.	VE	FMA, FIM	BF, ACT
Piron, 2009 [23]	VE tele-rehab vs. CT	VR with telemedicine (VRRS.net) for upper limb training. Therapist supported through videoconferencing.	Conventional UL therapy progressing in complexity from postural control to postural control with complex motion.	VE	FMA, Ashworth Scale	BF, BF
Piron, 2010 [46]	VE therapy vs. CT	Reinforced feedback in VR environment for UL training with therapist.	Conventional UL therapy progressing in complexity with PT.	VE	FMA, FIM	BF, ACT
Saposnik, 2010 [16]	Wii games + Standard therapy vs. table top games + Standard therapy	VR Wii therapy targeting UL. Co-intervention: Conventional OT & PT 1 hr each per day.	Leisure activities, such as playing cards, Bingo, or Jenga. Co-intervention: Conventional OT & PT 1 hr each per day.	CG	WMFT, BBT, SIS (hand items)	ACT, ACT, BF
Subramanian, 2013 [27]	VE training vs. physical training	VR based UL training (reaching for 6 targets).	Reaching for 6 targets in non-VR environment.	VE	WMFT, RPSS (close, far items)	ACT, BF, BF
Yang, 2008 [31]	VE treadmill vs. treadmill	VR based treadmill training designed to simulate typical community in Taipei (lane walking, street crossing, stepping over obstacles).	Treadmill training while executing different tasks (lifting legs to simulate walking over obstacles, uphill, downhill and fast walking).	VE	Gait speed, walking time in community	BF, ACT
Yavuzer, 2008 [17]	Playstation EyeToy games + Standard therapy vs. sham + Standard therapy	Playstation EyeToy games targeting UL movements. Co-intervention: Conventional OT, PT, and SLP.	Watched Playstation EyeToy games but did not play. Co-intervention: Conventional OT, PT, and SLP.	CG	FIM (self care items), Brunnstrom stages (hand, UE items)	ACT, BF, BF
You, 2005 [47]	VE exercise games vs. CT	IREX VR system targeting range of motion, balance, mobility, stepping and ambulation.	No treatment.	VE	FAC, MMAS (walking items)	ACT, ACT

Abbreviations: ACT, activity; ARAT, Action Research Arm Test; BBA, Brunel Balance Assessment; BBS, Berg Balance Scale; BBT, Box and Block Test; BF, body function; CAHAI, Chedoke Arm and Hand Activity Inventory; CG, commercial gaming; CT, conventional therapy; FES, Functional Electrical Stimulation; FIM, Functional Independence Measure; FMA, Fugl-Meyer Assessment; ICF, International Classification of Function, Disability, and Health; JTHF, Jebsen-Taylor Hand Function Test; MBI, Modified Barthel Index; MFT, Manual Function Test; MMAS, Modified Motor Assessment Scale; MSS, Motor Status Scale; MVPT, Motor-free Visual Perception Test; OT, occupational therapy; PART, participation; PASS, Postural Assessment Scale; PT, physiotherapy; RA, robotic assisted therapy; RPSS, Reaching Performance for Stroke Scale; SIS, Stroke Impact Scale; SLP, speech and language therapy; TUG, Time Up-and-Go test; UL, upper limb; VE, virtual environments; VR, virtual reality; WMFT, Wolf Motor Function Test; 10mWT, 10-metre Walk Test.

*  =  control group was not matched for time to the experimental group.

### Search Strategy

Relevant literature was first identified through electronic searches. A liaison librarian within the Faculty of Medicine at the University of British Columbia was consulted in selecting appropriate databases and developing the search strategy, including identifying key words and medical subject headings (MeSH terms). On April 4, 2013, electronic searches were conducted from the earliest available date in Medline, CINAHL, EMBASE, ERIC, PSYCInfo, DARE, PEDro, the Cochrane Central Register of Controlled Trials, and the Cochrane Database of Systematic Reviews. Population search terms were restricted to stroke and stroke synonyms, and intervention search terms included "video game", "virtual reality", and "augmented reality". Further relevant articles were identified by manually searching the bibliographies of retrieved papers. See Appendix S1 for the full search strategy.

### Study Selection

**Figure 1:**
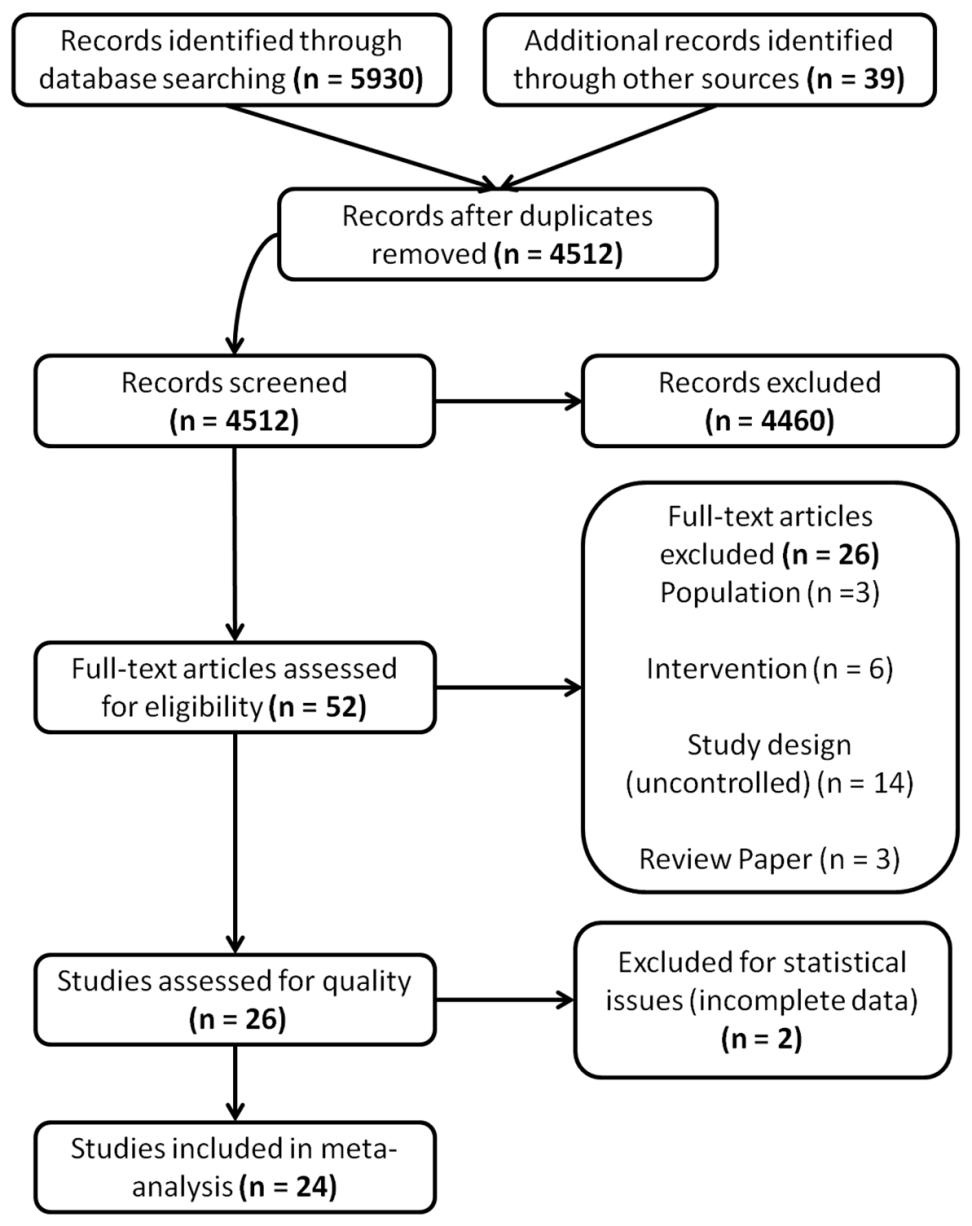
Screening of articles. Four-phase PRISMA flow-diagram for study collection [24], showing the number of studies identified, screened, eligible, and included in the review and analysis.

Following removal of duplicate publications, 4512 records were screened for eligibility (See Figure 1). The following exclusion criteria were used to screen the studies: (a) studies of children (<18 years old), (b) studies where fewer than 70% of subjects were adults post-stroke (e.g., studies involving cerebral palsy, traumatic brain injury, and other neurological disorders were excluded), (c) studies that did not use CT control conditions (e.g., studies comparing robotic assistance in combination with virtual reality to robotic assistance alone were excluded), (d) studies that did not use randomization or quasi-randomization with an appropriate control (e.g., case reports, case series, and uncontrolled trials were excluded), and (e) studies not published or translated into English were not searched. (Note, non-English studies were not excluded, but only studies published in English or translated into English were searched. Thus, relevant non-English studies may exist, but were not included, in our search. Despite this last criterion, the pool of included studies was highly international with studies from Canada, USA, Japan, Taiwan, Sweden, Italy, and Brazil.)

One author (CH) screened articles by title and abstract according to these criteria. Next, four authors used these criteria to screen the remaining articles by full text for inclusion. When there was disagreement, authors discussed the articles in question until consensus was reached. A total of 26 trials remained and were included in the assessment of study quality, but two of these articles were subsequently excluded for a lack of necessary data [25],[26], leaving 24 randomized controlled trials (RCTs) in the quantitative analysis.

### Quality Assessment

Three authors (CH, KC, ST) assessed the methodological quality of individual studies using the Physiotherapy Evidence Database Scale (PEDro; www.pedro.org.au), a criterion based measure of quality for randomized controlled trials. PEDro assesses 11 criteria to determine the selection, performance, detection, and attrition biases present within a study. For this review's quality assessment, a sample of 5 studies was extracted and all authors provided ratings. Across the 5 studies and 11 items of the PEDro Scale, reviewers had 93% initial agreement. Differences were discussed until 100% agreement was reached and authors proceeded to independently code the remaining studies.

### Quantitative Analysis

Three authors (CH, KC, ST) extracted data relevant to sample size, participant characteristics, intervention protocols, and outcome measures. One author (KL) extracted initial statistical data. All statistical data were then corroborated by an additional author; CH, KC, or ST. All calculations were based on data in the published manuscript except in one case [27], where additional data was requested and subsequently provided from the original authors.

Multiple outcome variables from each study were extracted in order to conduct separate analyses for each ICF category (see Table 1). Because the dependent measures fell into three ICF categories (viz. body function, activity, participation), each study had to contribute at least one and no more than three outcome variables. Outcomes were selected based on ICF category, and then precedence was given to primary outcomes. Thus, a study could report a body function outcome, an activity outcome, and a participation outcome. Or, if a study reported activity outcomes and two body function outcomes, the body function outcomes would be averaged together to create a single standardized effect size. This method was selected because it allows multiple outcomes to be selected from each study up to the maximum of one participation, one activity, and one body function outcome, or a maximum of three outcome measures from a single study (if not all ICF categories were measured).

Means, standard deviations, and sample sizes for the experimental group and the control group were entered into an Excel 2010 (Microsoft) spreadsheet and standardized effect-sizes (Hedge's G) and effect-size variability (V_G_) were calculated according to Borenstein, Hedges, Higgins, and Rothstein [28]. Effect-size calculations were arranged such that effects favouring VR therapy always had a positive value and effects favouring CT had a negative value. An effect size of zero indicating no difference between VR and CT. (The full dataset is provided in Appendix S2.) Effect-size measures and demographic information were imported into the statistical analysis software R (cran.r-project.org) and analyzed using the "metafor" package [29]. Custom scripts (Appendix S3) were written to test random-effects models for the overall effect of VR therapy compared to CT and meta-regression models to explore the influence of moderator variables on any VR therapy advantage. In these regressions, we tested the effect VR therapy type (VE versus CG) and the effect of time (in years) from stroke to onset of intervention.

## Results

Of the 24 VR studies included in the quantitative analysis, only four studies (16.7%) used CG [14]–[17] and the remaining 20 studies (83.3%) used VE. Often, studies used these VEs in conjunction with another apparatus, such as simulated environments during treadmill walking [30],[31]. In four studies (16.7%) [32]–[35], confounding conditions were present in the experimental methods. In these studies, experimental groups received VE therapy in addition to CT whereas the control group received CT alone, without being matched for time. Consequently, in these four studies, it remains unclear how much of the benefit of therapy can be attributed to the VE versus the additional time in therapy. With respect to the ICF categories that were explored, 32 outcome variables were measures of activity; 24 were measures of body function; and three were measures of participation. See Table 1.

### Methodological Quality

PEDro scores for the various studies were moderate, with a mean of 5.42 and SD of 1.60. The number of studies meeting each PEDro criterion is shown in Table 2. Studies generally met criteria for explicitly stating patients' eligibility (88.5%), random allocation to groups (84.6%), statistical comparisons of treatment and control groups (84.6%), and providing means/SDs for important variables (96.2%). A moderate number of studies met criteria for blinding of assessors (61.5%), achieving follow-up assessments for more than 85% of study participants (76.9%), and having comparable groups determined by baseline measurements (61.5%).

**Table 2:** Studies that meet the criteria of the PEDro scale.

			Selection Bias			Performance Bias		Detection Bias	Attrition Bias			
First Author	Year	C1	C2	C3	C4	C5	C6	C7	C8	C9	C10	C11
Broeren	2008	1	0	0	1	0	0	0	1	0	0	1
Cho	2013	1	1	1	1	0	0	1	1	0	1	1
Cikajilo	2012	1	0	0	0	0	0	1	1	0	1	1
Crosbie	2008	1	1	0	1	0	0	1	1	1	1	0
Crosbie	2012	1	1	1	1	0	0	1	1	1	1	1
da Silva Camiero	2011	1	1	0	0	0	0	1	0	0	1	1
Gil-Gómez	2011	1	1	0	1	0	0	1	1	0	1	1
In	2012	1	1	0	1	0	0	0	0	0	1	1
Jung	2012	1	1	0	0	1	0	1	1	0	1	1
Katz	2005	0	0	0	1	0	0	0	1	0	1	1
Kihoon	2012	1	1	1	1	1	0	0	1	1	0	1
Kim	2011	1	1	0	1	0	0	1	1	0	1	1
Kim	2012	1	1	0	0	0	0	0	0	0	1	1
Kiper	2011	1	1	0	1	0	0	0	1	1	1	1
Kwon	2012	1	1	0	1	1	1	1	1	0	1	1
Lam	2006	1	1	1	1	1	0	0	1	0	0	1
Mirelman	2010	0	1	0	0	1	0	0	1	0	1	1
Piron	2007	0	0	0	0	0	0	0	1	0	0	1
Piron	2009	1	1	1	0	0	0	1	1	0	1	1
Piron	2010	1	1	1	0	0	0	1	1	1	1	1
Saposnik	2010	1	1	0	0	0	0	1	0	0	1	1
Subramanian	2013	1	1	1	1	0	0	1	1	0	1	1
Yang	2008	1	1	1	1	0	0	1	0	0	1	1
Yang	2011	1	1	0	0	0	0	1	0	0	1	1
Yavuzer	2008	1	1	1	1	0	0	1	1	0	1	1
You	2005	1	1	0	1	0	0	0	1	0	1	1

Note. A "1" indicates that a study met that particular criterion, a "0" indicates that a study did not meet that criterion or that not enough information was given to make an assessment. C1  =  Eligibility criteria were specified; C2  =  Participants were randomly allocated to groups; C3  =  Treatment allocation was concealed; C4  =  Groups were similar at baseline; C5  =  Blinding of participants; C6  =  Blinding of therapists administering treatment; C7  =  Blinding of assessors for outcome measures; C8  =  Measurement of key outcome from >85% of participants; C9  =  Intention to treat analysis; C10  =  Between-groups statistical comparison is reported for key outcome; C11  =  Measures of central tendency and variability are provided. As per PEDro guidelines, the "total" score is based on C2 through C11.

Areas of weakness across studies were concealment of participant allocation (34.6%), blinding of participants (19.2%) and therapists (3.8%) to conditions, and following an intention to treat (ITT) analysis (19.2%). Proper concealment and ITT analysis are particularly important considerations; studies may have actually fulfilled these criteria but lacked explicit description in their Methods sections. Lack of blinding for both participants and therapists was also a limitation of the studies. Although it is not feasible to truly "blind" participants to the fact that they are receiving VR therapy, keeping patients and therapists naive to the experimental hypotheses would be a useful step to add experimental rigour and should be reported if it was achieved. A step further would be to use control conditions that also control for the social context or novelty of the VR therapy. For example, Saposnik and colleagues [16] compared Wii games and CT to a control group who engaged in tabletop games and CT, in an effort to control for the novelty, cognitive demands, and social context of the gaming intervention. Future research should attempt similar controls; the exact nature of these control groups would be dependent on the intervention.

### Demographic Characteristics of Included Studies

Sample sizes were quite small in the included studies, ranging from 5 to 40 participants per group (median was 11 participants per group; see Table 3). The intensity (min/day), frequency (days/week) and duration (weeks) of the interventions varied considerably. Interventions across studies ranged from 20-minute sessions [26],[39] to two-five hours of therapy per day (combined VR therapy, occupational and physical therapy) at frequencies of three to five sessions per week [17], and durations from two [16] to 12 weeks [39]. Multiplying intensity × frequency × duration yields total time scheduled for therapy in minutes. For total time, the shortest time scheduled for the VR therapy was 180 min [31] and the longest was 1800 min [45] (the median was 570 min). There was also considerable variability in the average years post-stroke for each study. The shortest average latency between stroke and study onset was 0.04 years [39] and the longest was 6.02 years [31] (the median was 1.05 years). The minimum average age for participants in these studies was 47.45 years and the maximum was 71.37 years (median average age was 61.30 years).

**Table 3:** Demographic statistics for the included studies.

Reference	VR Type	Time Scheduled for VR Intervention (min)	Experimental Group N	Control Group N	Years Post-Stroke (average)	Average Patient Age (yrs)
Broeren, 2008 [32]	VE	45*3*4 = 540‡	11	11	5.87	NR; range: 44-85
Cho, 2013 [36]	VE	30*3*6 = 540	7	7	0.82	64.85
Cikajlo, 2012 [37]	VE	20*5*3 = 300	6	20	0.36	58.50
Crosbie, 2012 [38]	VE	37.5*3*3 = 337.5	9	9	0.90	60.35
da Silva Cameirao, 2011 [39]	VE	20*3*12 = 720	8	8	0.04	61.37
Gil-Gómez, 2011 [15]	CG	60*20 sessions = 1200	9	8	1.58	47.45
In, 2012 [40]	VE	30*5*4 = 600	11	8	1.11	63.97
Jung, 2012 [30]	VE	30*5*3 = 450	11	10	1.17	62.05
Katz, 2005 [41]	VE	45*3*4 = 540	11	8	0.11	62.85
Kihoon, 2012 [34]	VE	30*3*4 = 360‡	15	14	NR	63.85
Kim, 2009 [33]	VE	30*4*4 = 480‡	12	12	0.07	52.09
Kim, 2012 [14]	CG	30*3*3 = 270	10	10	1.05	48.15
Kiper, 2011 [42]	VE	60*5*4 = 1200	40	40	0.48	64.00
Kwon, 2012 [35]	VE	30*5*4 = 600‡	13	13	0.67	57.54
Lam, 2006 [43]	VE	NR	20	16	4.74	71.37
Mirelman, 2010 [44]	VE	60*3*4 = 720	9	9	>2.00†	62.00
Piron, 2007 [45]	VE	60*5*6 = 1800	25	13	0.22	61.50
Piron, 2009 [23]	VE	60*5*4 = 1200	18	18	1.11	65.20
Piron, 2010 [46]	VE	60*5*4 = 1200	27	20	1.27	60.50
Saposnik, 2010 [16]	CG	60*8 sessions = 480	9	9	0.07	61.30
Subramanian, 2013 [27]	VE	45*3*4 = 540	16	16	3.35	61.00
Yang, 2008 [31]	VE	20*3*3 = 180	9	11	6.01	58.17
Yavuzer, 2008 [17]	CG	30*5*4 = 600	10	10	0.33	61.10
You, 2005 [47]	VE	60*5*4 = 1200	5	5	1.57	57.10

Note. Time scheduled for the VR intervention is given as (min/day) * (days/week) * (weeks)  =  total time in minutes. NR  =  ‘not reported’.

† =  this study did not report an average time post-stroke, so the minimum time was used instead.

‡ =  control group was not matched for time to the experimental group.

### Meta-Analysis: ICF Categories

In order to quantify effects of VR therapy we conducted separate random-effects meta-analyses for each ICF category. Separate analyses were used to ensure that different outcomes from the same study were analyzed independently. When studies had multiple outcomes within the same category (e.g., two activity outcomes) these effect-sizes were averaged together. Thus, each study contributed one data-point (at most) to the body function analysis, the activity analysis, and the participation analysis.

#### Body Function Outcomes: VE and CG Combined

For body function outcomes combining VE and CG interventions, the overall Hedge's G = 0.48, 95% Confidence Interval = [0.27, 0.70], which was significant, Z_obs_ = 4.33, P<0.001. The random-effects model, estimated using restricted maximum likelihood, had a τ^2^ = 0.05 (which is the estimate of variance between effects), I^2^ = 24.79% (which is the % of total variability due to heterogeneity), and H^2^ = 1.33 (which is the proportion of total variability to sampling variability). The test for heterogeneity was not significant, Q(15) = 21.55, P = 0.12. We tested years post-stroke as a potential moderating factor, but time post-stroke did not significantly affect outcomes (P = 0.76). We also tested the type of VR therapy used as a moderating factor (CG interventions were coded as 0 and VE interventions were coded as 1 in the regression), but type of therapy did not significantly affect outcomes (P = 0.38). Thus, there was an overall benefit of VR therapy for body function outcomes in adults post-stroke and we found no evidence that this effect was attenuated by the time post-stroke or the type of therapy given. Individual analyses for VE and CG studies are provided below.

#### Body Function Outcomes: Virtual Environments

**Figure 2:**
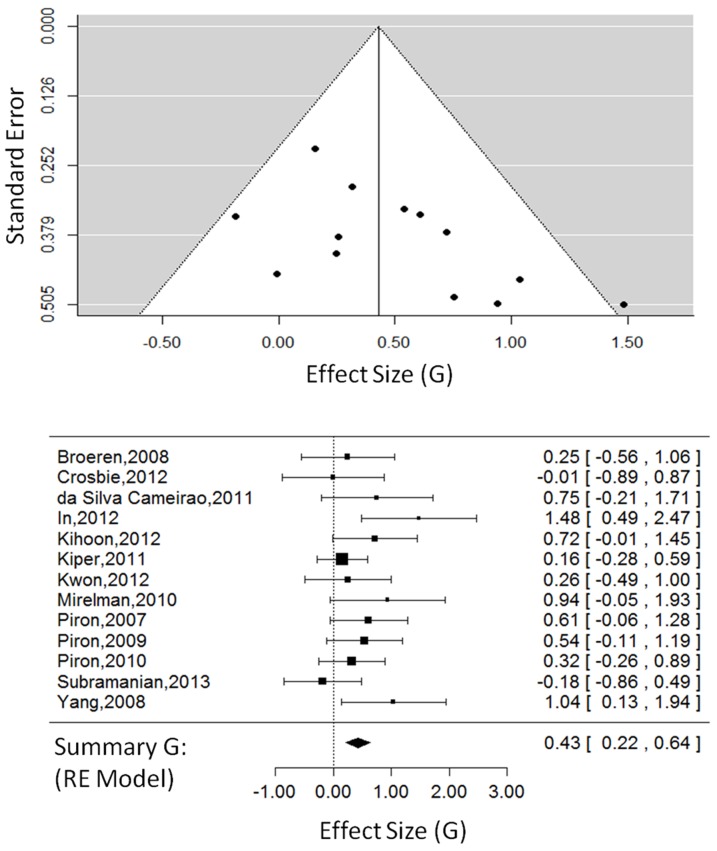
Body function outcomes in VE studies. The funnel plot (top) for body function outcomes showing effect-sizes (G) as a function of precision (standard error) in each virtual environment study. The forest plot (bottom) showing the effect-sizes and 95% confidence intervals for each study and the summary effect-size from the random-effects model. Positive values show a difference in favour of VE therapy. Negative values show a difference in favour of CT. Abbreviations: VE, virtual environments; RE, random effects.

For VE studies only (13 studies, 401 total participants, see Figure 2), the overall effect size was G = 0.43, 95% CI = [0.22, 0.64], which was significant, Z_obs_ = 3.97, P<0.001. The random-effects model, estimated using restricted maximum likelihood, had a τ^2^ = 0.02, I^2^ = 11.03%, and H^2^ = 1.12. The test for heterogeneity was not significant, Q(12) = 14.64, P = 0.26.

#### Body Function Outcomes: Commercial Games

**Figure 3:**
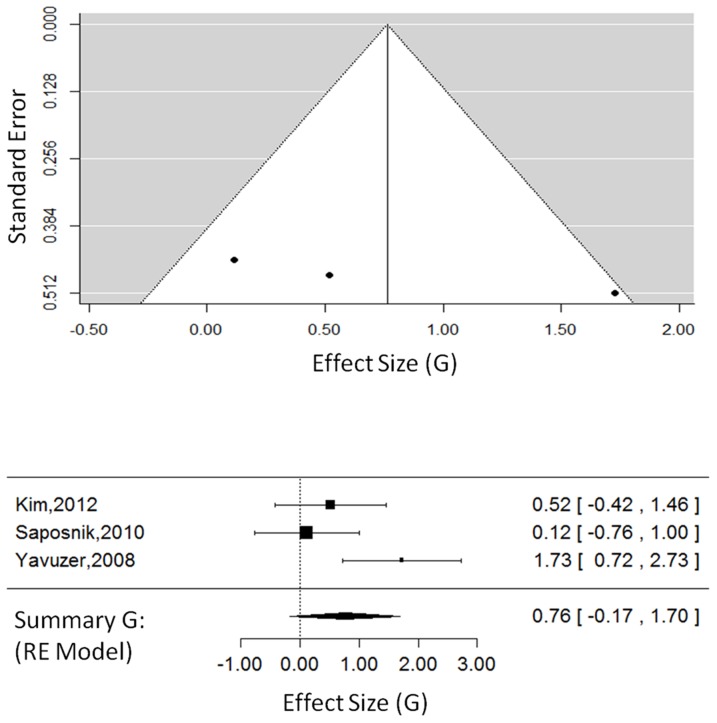
Body function outcomes in CG studies. The funnel plot (top) for body function outcomes showing effect-sizes (G) as a function of precision (standard error) in each commercial gaming study. The forest plot (bottom) showing the effect-sizes and 95% confidence intervals for each study and the summary effect-size from the random-effects model. Positive values show a difference in favour of CG therapy. Negative values show a difference in favour of CT. Abbreviations: CG, commercial gaming; RE, random effects.

For CG studies only (3 studies, 58 total participants, see Figure 3), the overall effect size was G = 0.76, 95% CI = [−0.17, 1.70], which approached significance, Z_obs_ = 1.60, P = 0.10. The random-effects model, estimated using restricted maximum likelihood, had a τ^2^ = 0.45, I^2^ = 66.30%, and H^2^ = 2.97. The test for heterogeneity approached significance, Q(2) = 5.85, P = 0.05.

#### Activity Outcomes: VE and CG Combined

For activity outcomes, the overall effect size was G = 0.58, 95% CI = [0.32, 0.85], which was significant, Z_obs_ = 4.32, P<0.001. The random-effects model had a τ^2^ = 0.21, I^2^ = 55.23%, and H^2^ = 2.23. The test for heterogeneity was significant, Q(21) = 49.18, P<0.01, thus there was significantly more variability in activity outcomes than would be predicted by sampling variability alone. Again, we tested time post-stroke as a moderating factor, but it was not significant (P = 0.65). We also tested the type of VR therapy used a moderating factor, but type of therapy did not significantly affect outcomes (P = 0.66). Thus, there was an overall benefit of VR therapy for activity outcomes in adults post-stroke and we found no evidence that this effect was attenuated by the time post-stroke or the type of therapy given. Individual analyses for VE and CG studies are provided below.

#### Activity Outcomes: Virtual Environments

**Figure 4:**
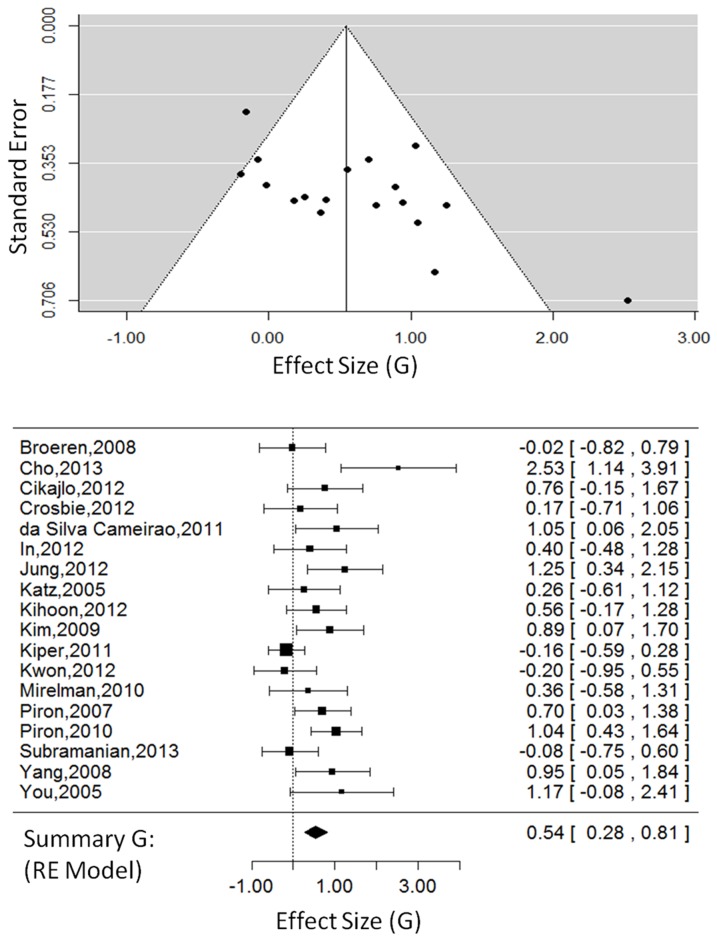
Activity outcomes in VE studies. The funnel plot (top) for activity outcomes showing effect-sizes (G) as a function of precision (standard error) in each virtual environment study. The forest plot (bottom) shows the effect-sizes and 95% confidence intervals for each study and the summary effect-size from the random-effects model. Positive values show a difference in favour of VE therapy. Negative values show a difference in favour of CT. Abbreviations: RE, random effects.

For VE studies only (18 studies, 479 total participants, see Figure 4), the overall effect size was G = 0.54, 95% CI = [0.28, 0.81], which was significant, Z_obs_ = 4.00, P<0.001. The random-effects model, estimated using restricted maximum likelihood, had a τ^2^ = 0.15, I^2^ = 49.18%, and H^2^ = 1.96. The test for heterogeneity was significant, Q(17) = 35.99, P<0.01.

#### Activity Outcomes: Commercial Gaming

**Figure 5:**
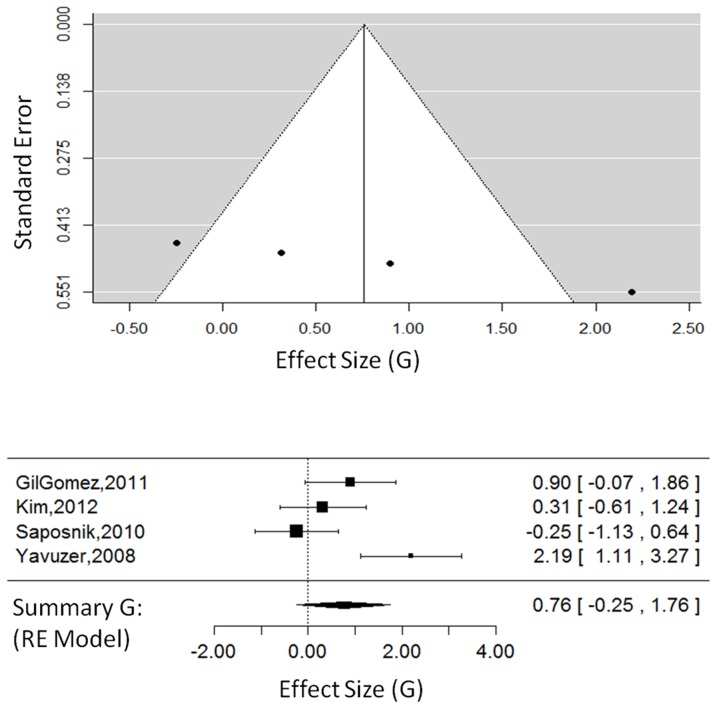
Activity outcomes in CG studies. The funnel plot (top) for activity outcomes showing effect-sizes (G) as a function of precision (standard error) in each commercial gaming study. The forest plot (bottom) shows the effect-sizes and 95% confidence intervals for each study and the summary effect-size from the random-effects model. Positive values show a difference in favour of CG therapy. Negative values show a difference in favour of CT. Abbreviations: CG, commercial gaming; RE, random effects.

For CG studies only (4 studies, 75 total participants, see Figure 5), the overall effect size was G = 0.76, 95% CI = [−0.25, 1.76], which was not significant, Z_obs_ = 1.48, P = 0.14. The random-effects model, estimated using restricted maximum likelihood, had a τ^2^ = 0.80, I^2^ = 77.17%, and H^2^ = 4.38. The test for heterogeneity was significant, Q(3) = 12.56, P<0.01.

#### Participation Outcomes

**Figure 6:**
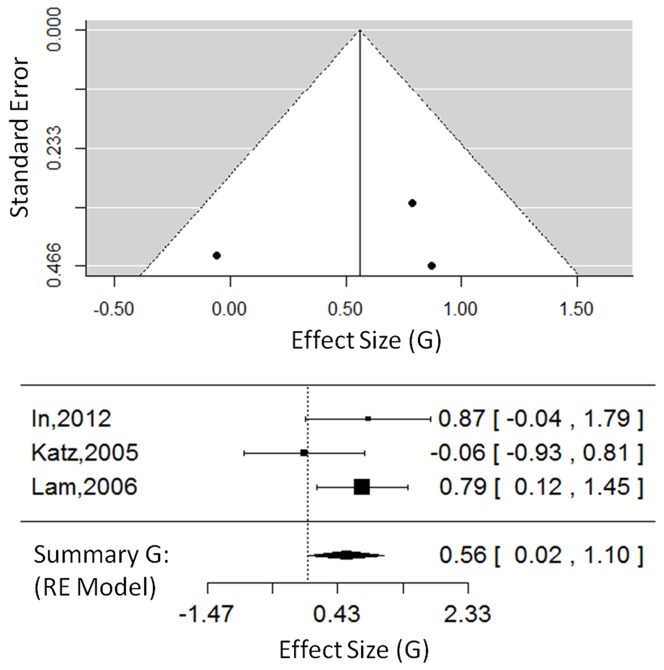
Participation outcomes in VE studies. The funnel plot (top) for participation outcomes showing effect-sizes (G) as a function of precision (standard error) in each study. The forest plot (bottom) shows the effect-sizes and 95% confidence intervals for each study and the summary effect-size from the random-effects model. Positive values show a difference in favour of VE therapy. Negative values show a difference in favour of CT. Abbreviations: VE, virtual environments; RE, random effects.

For participation outcomes (3 studies, 74 total participants, see Figure 6), the overall effect size was G = 0.56, 95% CI = [0.02, 1.10], which was significant, Z_obs_ = 2.02, P = 0.04. The random-effects model had a τ^2^ = 0.06, I^2^ = 26.75%, and H^2^ = 1.37. The test for heterogeneity was not significant, Q(2) = 2.82, P = 0.24. Also, given the small number of studies in this category, these results should be interpreted with caution. Due to the lack of sufficient data points, we were unable to test the moderating effect of time post-stroke or the effects of different VR interventions (all participation outcomes came from studies using VE interventions). These findings provide preliminary evidence that VR therapy has a positive effect on participation outcomes, but this is an understudied area of research, and more participation outcomes should be included in future studies.

## Discussion

This meta-analysis and systematic review is the first to examine the effects of VR across levels of the ICF and to compare effect-sizes as a function of the type of VR therapy implemented. These findings build upon previous reviews that have explored VR therapy compared to CT in general. This review adds to the current body of literature in three key areas: (1) 14 new RCTs have been published since previous reviews and are included in our analysis; (2) this review found positive effects of VR therapy across domains of the ICF; and (3) VR therapies were found to be effective when delivered as VE or CG. In the current analysis, time post-stroke and the type of VR intervention were not found to significantly affect outcomes. However, the small number of CG studies all had poor precision (shown in Figures 3 and 5), so larger trials with carefully designed control groups using CG interventions are needed before conclusions can be drawn about the efficacy of CG interventions.

### Review identifies new trials

The most recent previous reviews summarizing the evidence for VR therapy included searches of the literature up to March and July 2010 [19],[20]. Fourteen trials (58.3%) included in our review were published after 2010 and had not yet been included in a meta-analysis. Furthermore, previous reviews [19] included observational studies, whereas we selected only randomized controlled trials to ensure robustness of the evidence. All previous reviews of this topic have demonstrated a moderate effect in favour of VR therapy over CT [19]–[21] however, the heterogeneity of trial parameters requires that all effect sizes and conclusions be interpreted with caution. Similarly, our review suggests VR therapy has a moderate effect on outcomes for adults after stroke, but many sources of variability exist in the interventions and outcomes of the included trials.

### Sources of variability within interventions

There was considerable variability in how VR interventions were delivered with respect to intensity, frequency and duration of the intervention. VR interventions were also inconsistently conducted in conjunction with other PT/OT treatments. As such, we are unable to comment on optimal prescribing dosage for VR therapies.

Studies lacked detail about the content of the CT being compared to VR. Studies were inconsistent in their reporting of the role(s) of therapists, rehabilitation assistants, caregivers, and/or other personnel; future research should ensure sufficient information is given to readers to allow for accurate comparisons. Individual studies did often schedule equal time in therapy for experimental and control groups, but most studies did not ensure true dosage matching of groups (e.g., matching active time in therapy or numbers of repetitions). Subramanian and colleagues [27] explicitly matched arm-reaching repetitions between the experimental and control groups, and they also controlled for the amount of feedback (knowledge of results and performance) provided. Repetitions were controlled in that study, and there was no overall benefit of VR therapy beyond CT. However, VR training did lead to larger improvements in participants with mild impairments compared to CT, and VR training reduced compensatory movements in moderate-to-severely impaired participants compared to CT. Future studies should use similar methods for controlling repetitions when investigating VR therapies to clarify our understanding of the benefits of VR in therapy.

Another source of variability could be the degree to which participants felt motivated and engaged during therapy. It has been suggested that VR therapies are advantageous to CT in part because of the motivating influence of using novel technologies or games [10],[11]. Unfortunately, most of the trials included in this review do not discuss motivation, use motivation as an outcome measure, or control for the motivating or novel components of VR therapy. Some studies did attempt to control for this factor using card games [16] or cognitive computer games [33] in their control group. Arguably, interactive video games may still be considered more novel to an older population.

### No differences found for VR therapy types

A major objective of our review was to compare the effects of commercial gaming systems (CG) to rehabilitation-specific virtual environments (VE) in a therapy context for adults post-stroke. We found no evidence for differences between VE and CG games in the current analysis, but CG interventions have been too few and too small to draw conclusions. Four trials (16.7%) examined the effects of CG therapy [14]–[17] and 20 trials (83.3%) researched VE therapy compared to CT for adults post-stroke. Our meta-analysis provides strong evidence for the effectiveness of VE interventions and demonstrates promising initial data for the effectiveness of CG interventions. More data needs to be collected to see if gains for CG interventions are reliable and to see if the moderate effect-sizes observed (from G = 0.4–0.7) translate into clinically meaningful results. These results suggest larger RCTs using CG interventions are justified; we recommend RCTs compare CG directly to VE and CT groups.

Movement-controlled games are increasingly investigated as therapeutic tools for individuals with neurological disorders such as cerebral palsy [48] and stroke [49]. An appealing aspect of movement-controlled games is combining aerobic exercise and motor skills practice, which may increase neuroplasticity during motor rehabilitation [50]. As a result, commercial games have been investigated as tools for learning motor skills and for improving cardiovascular fitness. For example, the game Dance Dance Revolution has been shown to increase energy expenditure in adolescents up to 5.4 (1.8 SD) Metabolic Equivalent Tasks (METs) [51]. In healthy adults, Wii Sports tennis requires 2.1 (1.2 SD) METs, baseball 2.8 (0.9 SD) METs, and boxing 4.7 (1.4 SD) METs [48]. However, in adults with cerebral palsy, the same games all increase energy expenditure to over 3 METs [48], suggesting they can help these individuals meet recommended guidelines for physical activity. In addition to increasing energy expenditures, commercial movement games have been used therapeutically to improve balance, strength and coordination [49],[52].

Increased availability and lower cost are also potential advantages of using commercial games over virtual reality systems that have been designed specifically for rehabilitation. For example, the Nintendo Wii (Nintendo Co., Kyoto, JP) has been sold to over 100 million customers worldwide [53], and the console retails below US$150. As a comparison, the GestureTek IREX (GestureTek, Toronto, CA) system, used in the study by Kwon et al. in 2012 [35], is only available through specialized rehabilitation equipment distributors and retails at more than US$15,000 [54]. Understanding the benefits of CG and VE systems, relative to their costs, thus has significant implications for therapists and clients facing budget constraints.

### Positive effects of VR therapy across ICF categories

The ICF provides a framework and a comprehensive perspective of functioning and disability in research and clinical practice [55]. The overarching goal of rehabilitation for adults post-stroke is to restore the person's ability to participate in normal life roles with as much independence as possible. Impairments at the body structure and function level may influence activity limitations, and activity limitations may influence participation restrictions [56]. However, impairments and activity limitations do not necessarily affect the enjoyment of participation by individuals in various life situations [57],[58]. It is, therefore, important for researchers and clinicians to be clear about which ICF domains an intervention intends to, and actually does, impact.

Our review did not identify any trials that examined outcomes related to body structures, personal factors, or environmental factors and only three trials (12.5%) that examined participation outcomes. There was a moderate but reliable advantage of VR therapy over CT in the categories of body function and activity, but outcomes from other ICF categories should be included in future research.

### Limitations

Our review included studies conducted in all stages of stroke recovery, from acute inpatient to chronic outpatient settings (average time post-stroke of the study participants ranged from 0.04 years [39] to 6.02 years [31]). However, our analysis was based on a small number of studies making statistical power a concern for these regression analyses. Furthermore, the small number of studies limits our ability to control for other moderating factors such as the initial severity of stroke and the effect of time post-stroke on conventional therapy outcomes.

This review is limited by some risk of publication bias in the included studies. Visual inspection of the funnel plots in the figures reveals highly positive studies with low precision for both activity and body function outcomes. For activity outcomes, two outlying studies (8.3%) [17],[36] had small numbers of subjects (N = 20 and 14, respectively) but relatively good study quality (7/10 for both studies on PEDro criteria). Similarly for body function outcomes, two studies (8.3%) [17],[40] had small numbers of subjects (N = 20 and 19, respectively) and while Yavuzer et al. [17] had good study quality (7/10), In et al. [40] had poor study quality (4/10).

## Conclusions

This review updated the evidence for virtual reality therapy to include the most recent trials, and is the first to investigate the effects of VR therapy across ICF domains and between VR therapy types. Virtual reality therapy demonstrates a significant moderate advantage in body function and activity outcomes when compared to CT. Research on participation outcomes is limited, but initial data show a positive benefit of VR therapy compared to CT. No significant differences were found between the VE and CG therapy types, and there was no evidence that time post-stroke attenuated the benefits of VR therapy, but these findings are limited by a high degree of variability between studies. To date, CG interventions have been too few and too small to draw strong conclusions about their efficacy. Larger RCTs investigating CG interventions would provide better evidence for their use in therapy as a potentially effective and cost-efficient method of increasing motor repetitions in a motivating way. Given the relationship between participation and quality of life, it is also recommended that future trials include participation outcome measures in their investigations.

## Supporting Information

Appendix S1 **Comprehensive search strategy used in the systematic review.**
(DOC)

Appendix S2 **Full data-set of effect-sizes and demographic information for each study.**
(XLSX)

Appendix S3 **Scripts for analysis.** Written in "R" using the "metafor" package.
(TXT)

Checklist S1 **PRISMA checklist.**
(DOC)
