# Supplementary material for: Virtual Reality Therapy for Adults Post-Stroke: A Systematic Review and Meta-Analysis Exploring Virtual Environments and Commercial Games in Therapy
Source: PLoS One. 2014 Mar 28;9(3):e93318. doi: 10.1371/journal.pone.0093318 (PMC3969329; doi:10.1371/journal.pone.0093318)
Supplement: Appendix S1 — Comprehensive search strategy used in the systematic review. (DOC) [file pone.0093318.s001.doc]

**Supplement 1**

Search Strategies – April 4, 2013 except Cinahl (Apr 8)

**MEDLINE (OvidSP) 1946 to Present**

1. cerebrovascular disorders/ or exp basal ganglia cerebrovascular disease/ or exp brain ischemia/ or exp carotid artery diseases/ or cerebrovascular accident/ or exp brain infarction/ or exp cerebrovascular trauma/ or exp hypoxia-ischemia, brain/ or exp intracranial arterial diseases/ or intracranial arteriovenous malformations/ or exp intracranial hemorrhages/ or vasospasm, intracranial/ or vertebral artery dissection/

2. (stroke or poststroke or post-stroke or cerebrovasc$ or brain vasc$ or cerebral vasc$ or cva$ or apoplex$ or SAH).tw.

3. ((brain$ or cerebr$ or cerebell$ or intracran$ or intracerebral) adj5 (isch?emi$ or infarct$ or thrombo$ or emboli$ or occlus$)).tw.

4. ((brain$ or cerebr$ or cerebell$ or intracerebral or intracranial or subarachnoid) adj5 (haemorrhage$ or hemorrhage$ or haematoma$ or hematoma$ or bleed$)).tw.

5. 1 or 2 or 3 or 4

6. Video Games/

7. (video gam* or computer gam*).mp. [mp=title, abstract, original title, name of substance word, subject heading word, keyword heading word, protocol supplementary concept, rare disease supplementary concept, unique identifier]

8. (wii or kinect or nintendo or playstation or xbox).mp. [mp=title, abstract, original title, name of substance word, subject heading word, keyword heading word, protocol supplementary concept, rare disease supplementary concept, unique identifier]

9. (augment* reality or virtual reality or augment* gam* or virtual gam*).mp [mp=title, abstract, original title, name of substance word, subject heading word, keyword heading word, protocol supplementary concept, rare disease supplementary concept, unique identifier]

10. 6 or 7 or 8 or 9

11. 5 and 10 **(310)**

**EMBASE (OvidSP) 1974 to 2013 April 05**

1. cerebrovascular disorders/ or exp basal ganglia cerebrovascular disease/ or exp brain ischemia/ or exp carotid artery diseases/ or cerebrovascular accident/ or exp brain infarction/ or exp cerebrovascular trauma/ or exp hypoxia-ischemia, brain/ or exp intracranial arterial diseases/ or intracranial arteriovenous malformations/ or exp intracranial hemorrhages/ or vasospasm, intracranial/ or vertebral artery dissection/

2. (stroke or poststroke or post-stroke or cerebrovasc$ or brain vasc$ or cerebral vasc$ or cva$ or apoplex$ or SAH).tw.

3. ((brain$ or cerebr$ or cerebell$ or intracran$ or intracerebral) adj5 (isch?emi$ or infarct$ or thrombo$ or emboli$ or occlus$)).tw.

4. ((brain$ or cerebr$ or cerebell$ or intracerebral or intracranial or subarachnoid) adj5 (haemorrhage$ or hemorrhage$ or haematoma$ or hematoma$ or bleed$)).tw.

5. 1 or 2 or 3 or 4

6. Video Games/

7. (video gam* or computer gam*).mp. [mp=title, abstract, subject headings, heading word, drug trade name, original title, device manufacturer, drug manufacturer, device trade name, keyword]

8. (wii or kinect or nintendo or playstation or xbox).mp. [mp=title, abstract, subject headings, heading word, drug trade name, original title, device manufacturer, drug manufacturer, device trade name, keyword]

9. (augment* reality or virtual reality or augment* gam* or virtual gam*).mp. [mp=title, abstract, subject headings, heading word, drug trade name, original title, device manufacturer, drug manufacturer, device trade name, keyword]

10. 6 or 7 or 8 or 9

11. 5 and 10 **(619)**

**DARE (OvidSP) 1st Quarter 2013**

1. cerebrovascular disorders/ or exp basal ganglia cerebrovascular disease/ or exp brain ischemia/ or exp carotid artery diseases/ or cerebrovascular accident/ or exp brain infarction/ or exp cerebrovascular trauma/ or exp hypoxia-ischemia, brain/ or exp intracranial arterial diseases/ or intracranial arteriovenous malformations/ or exp intracranial hemorrhages/ or vasospasm, intracranial/ or vertebral artery dissection/

2. (stroke or poststroke or post-stroke or cerebrovasc$ or brain vasc$ or cerebral vasc$ or cva$ or apoplex$ or SAH).tw.

3. ((brain$ or cerebr$ or cerebell$ or intracran$ or intracerebral) adj5 (isch?emi$ or infarct$ or thrombo$ or emboli$ or occlus$)).tw.

4. ((brain$ or cerebr$ or cerebell$ or intracerebral or intracranial or subarachnoid) adj5 (haemorrhage$ or hemorrhage$ or haematoma$ or hematoma$ or bleed$)).tw.

5. 1 or 2 or 3 or 4

6. Video Games/

7. (video gam* or computer gam*).mp. [mp=ti, ab, tx, kw, ct]

8. (wii or kinect or nintendo or playstation or xbox).mp. [mp=ti, ab, tx, kw, ct]

9. (augment* reality or virtual reality or augment* gam* or virtual gam*).mp. [mp=ti, ab, tx, kw, ct]

10. 6 or 7 or 8 or 9

11. 5 and 10 **(24)**

**Cochrane Database of Systematic Reviews (OvidSP) 2005 to February 2013**

1. cerebrovascular disorders/ or exp basal ganglia cerebrovascular disease/ or exp brain ischemia/ or exp carotid artery diseases/ or cerebrovascular accident/ or exp brain infarction/ or exp cerebrovascular trauma/ or exp hypoxia-ischemia, brain/ or exp intracranial arterial diseases/ or intracranial arteriovenous malformations/ or exp intracranial hemorrhages/ or vasospasm, intracranial/ or vertebral artery dissection/

2. (stroke or poststroke or post-stroke or cerebrovasc$ or brain vasc$ or cerebral vasc$ or cva$ or apoplex$ or SAH).tw.

3. ((brain$ or cerebr$ or cerebell$ or intracran$ or intracerebral) adj5 (isch?emi$ or infarct$ or thrombo$ or emboli$ or occlus$)).tw.

4. ((brain$ or cerebr$ or cerebell$ or intracerebral or intracranial or subarachnoid) adj5 (haemorrhage$ or hemorrhage$ or haematoma$ or hematoma$ or bleed$)).tw.

5. 1 or 2 or 3 or 4

6. Video Games/

7. (video gam* or computer gam*).mp. [mp=title, abstract, full text, keywords, caption text]

8. (wii or kinect or nintendo or playstation or xbox).mp. [mp=title, abstract, full text, keywords, caption text]

9. (augment* reality or virtual reality or augment* gam* or virtual gam*).mp.[mp=title, abstract, full text, keywords, caption text]

10. 6 or 7 or 8 or 9

11. 5 and 10 **(17)**

**Cochrane Central Register of Controlled Trials March 2013**

1. cerebrovascular disorders/ or exp basal ganglia cerebrovascular disease/ or exp brain ischemia/ or exp carotid artery diseases/ or cerebrovascular accident/ or exp brain infarction/ or exp cerebrovascular trauma/ or exp hypoxia-ischemia, brain/ or exp intracranial arterial diseases/ or intracranial arteriovenous malformations/ or exp intracranial hemorrhages/ or vasospasm, intracranial/ or vertebral artery dissection/

2. (stroke or poststroke or post-stroke or cerebrovasc$ or brain vasc$ or cerebral vasc$ or cva$ or apoplex$ or SAH).tw.

3. ((brain$ or cerebr$ or cerebell$ or intracran$ or intracerebral) adj5 (isch?emi$ or infarct$ or thrombo$ or emboli$ or occlus$)).tw.

4. ((brain$ or cerebr$ or cerebell$ or intracerebral or intracranial or subarachnoid) adj5 (haemorrhage$ or hemorrhage$ or haematoma$ or hematoma$ or bleed$)).tw.

5. 1 or 2 or 3 or 4

6. Video Games/

7. (video gam* or computer gam*).mp. [mp=title, original title, abstract, mesh headings, heading words, keyword]

8. (wii or kinect or nintendo or playstation or xbox).mp. [mp=title, original title, abstract, mesh headings, heading words, keyword]

9. (augment* reality or virtual reality or augment* gam* or virtual gam*).mp.[mp=title, original title, abstract, mesh headings, heading words, keyword]

10. 6 or 7 or 8 or 9

11. 5 and 10 **(52)**

**Cinahl with Full Text (EBSCO) 1981 to present** Searched April 8, 2013

S1 MM "cerebrovascular disorders+" or "cerebral ischemia+" or "basal ganglia cerebrovascular disease" or "carotid artery diseases" or "stroke" or "stroke patients" or "cerebral embolism" or "brain injuries" or "intracranial arterial diseases" or "intracranial arteriosclerosis" or "arteriovenous malformations" or "cerebral embolism" "thrombosis" or "intracranial haemorrhages"or "cerebral vasospasm" or "vertebral artery dissection"

S2 stroke or poststroke or post-stroke or cerebrovasc* or brain vasc* or cerebral vasc* or cva* or apoplexy* or SAH

S3 brain* or cerebr* or cerebell* or intracran* or intracerebral N5 isch?emi* or infarct* or thrombo* or emboli* or occlus*

S4 brain* or cerebr* or cerebell* or intracerebral or intracranial or subarachnoid N5 haemorrhage* or hemorrhage* or haematoma* or bleed*

S5 S1 OR S2 OR S3 OR S4

S6 (MH "Video Games")

S7 video gam* or computer gam*

S8 wii or kinect or nintendo or playstation or xbox

S9 augment* reality or virtual reality or augment* gam* or virtual gam*

S10 S6 OR S7 OR S8 OR S9

S11 S5 AND S10 **(346)**

**ERIC (EBSCO) 1966 to present**

S1 MM "cerebrovascular disorders+" or "cerebral ischemia+" or "basal ganglia cerebrovascular disease" or "carotid artery diseases" or "stroke" or "stroke patients" or "cerebral embolism" or "brain injuries" or "intracranial arterial diseases" or "intracranial arteriosclerosis" or "arteriovenous malformations" or "cerebral embolism" "thrombosis" or "intracranial haemorrhages"or "cerebral vasospasm" or "vertebral artery dissection"

S2 stroke or poststroke or post-stroke or cerebrovasc* or brain vasc* or cerebral vasc* or cva* or apoplexy* or SAH

S3 brain* or cerebr* or cerebell* or intracran* or intracerebral N5 isch?emi* or infarct* or thrombo* or emboli* or occlus*

S4 brain* or cerebr* or cerebell* or intracerebral or intracranial or subarachnoid N5 haemorrhage* or hemorrhage* or haematoma* or bleed*

S5 S1 OR S2 OR S3 OR S4

S6 (video gam* or computer gam*)

S7 (wii or kinect or nintendo or playstation or xbox)

S8 (augment* reality or virtual reality or augment* gam* or virtual gam*)

S9 S6 OR S7 OR S8

S10 S5 AND S9 **(71)**

**PSYCInfo (EBSCO) 1600s to present**

S1 MM "cerebrovascular disorders+" or "cerebral ischemia+" or "basal ganglia cerebrovascular disease" or "carotid artery diseases" or "stroke" or "stroke patients" or "cerebral embolism" or "brain injuries" or "intracranial arterial diseases" or "intracranial arteriosclerosis" or "arteriovenous malformations" or "cerebral embolism" "thrombosis" or "intracranial haemorrhages"or "cerebral vasospasm" or "vertebral artery dissection"

S2 stroke or poststroke or post-stroke or cerebrovasc* or brain vasc* or cerebral vasc* or cva* or apoplexy* or SAH

S3 brain* or cerebr* or cerebell* or intracran* or intracerebral N5 isch?emi* or infarct* or thrombo* or emboli* or occlus*

S4 brain* or cerebr* or cerebell* or intracran* or intracerebral N5 isch?emi* or infarct* or thrombo* or emboli* or occlus*

S5 S1 OR S2 OR S3 OR S4

S6 video gam* or computer gam*

S7 wii or kinect or nintendo or playstation or xbox

S8 augment* reality or virtual reality or augment* gam* or virtual gam*

S9 S6 OR S7 OR S8

S10 S5 AND S9 **(1202)**

**Compendex (EI Village) 1884 - present**

1. ((MM "cerebrovascular disorders+" or "cerebral ischemia+" or "basal ganglia cerebrovascular disease" or "carotid artery diseases" or "stroke" or "stroke patients" or "cerebral embolism" or "brain injuries" or "intracranial arterial diseases" or "intracranial arteriosclerosis" or "arteriovenous malformations" or "cerebral embolism" "thrombosis" or "intracranial haemorrhages"or "cerebral vasospasm" or "vertebral artery dissection") WN All fields)

2. ((stroke or poststroke or post-stroke or cerebrovasc* or brain vasc* or cerebral vasc* or cva* or apoplexy* or SAH) WN All fields)

3. ((brain* or cerebr* or cerebell* or intracran* or intracerebral N5 isch?emi* or infarct* or thrombo* or emboli* or occlus*) WN All fields)

4. ((brain* or cerebr* or cerebell* or intracerebral or intracranial or subarachnoid N5 haemorrhage* or hemorrhage* or haematoma* or bleed*) WN All fields)

5. ( ((brain* OR cerebr* OR cerebell* OR $intracerebral OR $intracranial OR $subarachnoid $N5 haemorrhage* OR hemorrhage* OR haematoma* OR bleed*) WN ALL) AND (1884-2013 WN YR)) OR ( ((brain* OR cerebr* OR cerebell* OR intracran* OR $intracerebral $N5 isch?emi* OR infarct* OR thrombo* OR emboli* OR occlus*) WN ALL) AND (1884-2013 WN YR)) OR ( (($stroke OR $poststroke OR $post-stroke OR cerebrovasc* OR $brain vasc* OR $cerebral vasc* OR cva* OR apoplexy* OR $SAH) WN ALL) AND (1884-2013 WN YR)) OR ( (($MM {cerebrovascular disorders+} OR {cerebral ischemia+} OR {basal ganglia cerebrovascular disease} OR {carotid artery diseases} OR{stroke} OR {stroke patients} OR {cerebral embolism} OR {brain injuries} OR {intracranial arterial diseases} OR {intracranial arteriosclerosis} OR {arteriovenous malformations} OR {cerebral embolism} {thrombosis} OR {intracranial haemorrhages} OR {cerebral vasospasm} OR {vertebral artery dissection}) WN ALL) AND (1884-2013 WN YR))

6. (((video gam* or computer gam*)) WN All fields)

7. (((wii or kinect or nintendo or playstation or xbox)) WN All fields)

8. (((augment* reality or virtual reality or augment* gam* or virtual gam*)) WN All fields)

9. ((((augment* $reality OR $virtual $reality OR augment* gam* OR $virtual gam*)) WN ALL) AND (1884-2013 WN YR)) OR ( ((($wii OR $kinect OR $nintendo OR $playstation OR $xbox)) WN ALL) AND (1884-2013 WN YR)) OR ( ((($video gam* OR $computer gam*)) WN ALL) AND (1884-2013 WN YR))

10. ((((((augment* $reality OR $virtual $reality OR augment* gam* OR $virtual gam*)) WN ALL) AND (1884-2013 WN YR)) OR (((($wii OR $kinect OR $nintendo OR $playstation OR $xbox)) WN ALL) AND (1884-2013 WN YR)) OR (((($video gam* OR $computer gam*)) WN ALL) AND (1884-2013 WN YR)))) AND ( ((((brain* OR cerebr* OR cerebell* OR $intracerebral OR $intracranial OR $subarachnoid $N5 haemorrhage* OR hemorrhage* OR haematoma* OR bleed*) WN ALL) AND (1884-2013 WN YR)) OR (((brain* OR cerebr* OR cerebell* OR intracran* OR $intracerebral $N5 isch?emi* OR infarct* OR thrombo* OR emboli* OR occlus*) WN ALL) AND (1884-2013 WN YR)) OR ((($stroke OR $poststroke OR $post-stroke OR cerebrovasc* OR $brain vasc* OR $cerebral vasc* OR cva* OR apoplexy* OR $SAH) WN ALL) AND (1884-2013 WN YR)) OR ((($MM {cerebrovascular disorders+} OR{cerebral ischemia+} OR {basal ganglia cerebrovascular disease} OR {carotid artery diseases} OR {stroke} OR {stroke patients} OR {cerebral embolism} OR {brain injuries} OR {intracranial arterial diseases} OR {intracranial arteriosclerosis} OR {arteriovenous malformations} OR {cerebral embolism}{thrombosis} OR {intracranial haemorrhages} OR {cerebral vasospasm} OR {vertebral artery dissection}) WN ALL) AND (1884-2013 WN YR)))) **(3253)**

**PEDro 1929 - Updated March 4, 2013**

Stroke and Virtual Reality **(31)**

Stroke and Augment* Reality **(3)**

Stroke and Video Game **(2)**
